# Point Mutations in Aβ Result in the Formation of Distinct Polymorphic Aggregates in the Presence of Lipid Bilayers

**DOI:** 10.1371/journal.pone.0016248

**Published:** 2011-01-18

**Authors:** Phillip M. Pifer, Elizabeth A. Yates, Justin Legleiter

**Affiliations:** 1 The C. Eugene Bennett Department of Chemistry, West Virginia University, Morgantown, West Virginia, United States of America; 2 WVnano Initiative, West Virginia University, Morgantown, West Virginia, United States of America; 3 The Center for Neurosciences, West Virginia University, Morgantown, West Virginia, United States of America; University of South Florida College of Medicine, United States of America

## Abstract

A hallmark of Alzheimer's disease (AD) is the rearrangement of the β-amyloid (Aβ) peptide to a non-native conformation that promotes the formation of toxic, nanoscale aggregates. Recent studies have pointed to the role of sample preparation in creating polymorphic fibrillar species. One of many potential pathways for Aβ toxicity may be modulation of lipid membrane function on cellular surfaces. There are several mutations clustered around the central hydrophobic core of Aβ near the α-secretase cleavage site (E22G Arctic mutation, E22K Italian mutation, D23N Iowa mutation, and A21G Flemish mutation). These point mutations are associated with hereditary diseases ranging from almost pure cerebral amyloid angiopathy (CAA) to typical Alzheimer's disease pathology with plaques and tangles. We investigated how these point mutations alter Aβ aggregation in the presence of supported lipid membranes comprised of total brain lipid extract. Brain lipid extract bilayers were used as a physiologically relevant model of a neuronal cell surface. Intact lipid bilayers were exposed to predominantly monomeric preparations of Wild Type or different mutant forms of Aβ, and atomic force microscopy was used to monitor aggregate formation and morphology as well as bilayer integrity over a 12 hour period. The goal of this study was to determine how point mutations in Aβ, which alter peptide charge and hydrophobic character, influence interactions between Aβ and the lipid surface. While fibril morphology did not appear to be significantly altered when mutants were prepped similarly and incubated under free solution conditions, aggregation in the lipid membranes resulted in a variety of polymorphic aggregates in a mutation dependent manner. The mutant peptides also had a variable ability to disrupt bilayer integrity.

## Introduction

**Figure 1:**
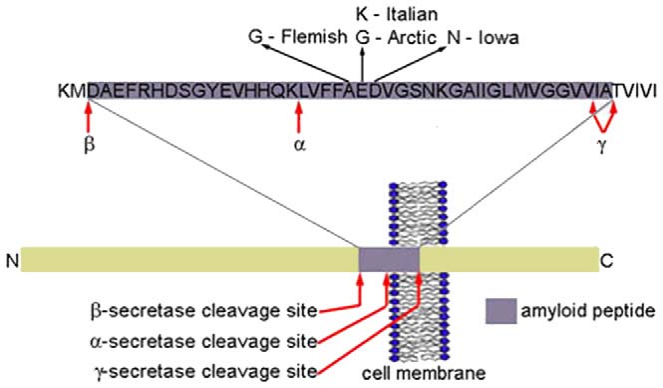
Schematic representation of APP processing and point mutations in Aβ. α-secretase-mediated proteolytic cleavage of APP occurs after residue 687 of APP, β-secretase-mediated cleavage occurs after residue 671, and γ-secretase cleavage at position 711 or 713. Successive cleavage by β-secretase and γ-secretase results in the release of an intact Aβ peptide. Several point mutations in APP and Aβ are indicated, including the Arctic, Italian, Iowa, and Flemish mutations, which were used in this study.

The neuropathological and neurochemical hallmarks of Alzheimer's disease (AD) include: synaptic loss and selective neuronal cell death; decreases in markers for certain neurotransmitters; and abnormalities in neurons and their processes (neurofibrillary tangles comprised of Tau and dystrophic neurites) as well as in the extracellular space (cerebrovascular, diffuse, and neuritic plaques - composed predominantly of the amyloidogenic peptide Aβ) [1], [2]. Aβ is formed by endoproteolytic cleavage of a single-transmembrane, receptor-like protein termed the β-amyloid precursor protein (APP) (Fig. 1). All AD patients develop neuritic plaques in brain regions subserving memory and cognition. These plaques consist of extracellular masses of Aβ filaments and other plaque associated proteins (e.g. apoE, apoJ, inflammatory molecules) which are intimately associated with dystrophic dendrites and axons, activated microglia, and reactive astrocytes [3].

It has been well established that many amyloid forming peptides have the ability to aggregate into a variety of morphologically distinct and stable fibril structures [4]. At a gross morphological level, this ability to form distinct polymorphic fibril structures of Aβ have been known for some time [5], [6]. By subtle alterations in fibril growth conditions, two structurally distinct polymorphic fibrils of Aβ (1–40) were formed that displayed significantly different levels of toxicity to neuronal cell cultures [7]. Another fibrillar polymorph of Aβ was identified by using fibrils extracted from AD brains as seeds [8]. Recent studies have demonstrated that variations in Aβ (1–40) sample preparation can result in at least five structurally-distinct fibrillar aggregates *in vitro* [9]. Collectively, these studies point to the notion that aggregate polymorphism may contribute to variations in AD.

A potential environmental factor influencing Aβ aggregate formation is the presence of lipid surfaces. In particular, it has been hypothesized that a potential pathway for Aβ toxicity may lie in its ability to modulate lipid membrane function. Thus, elucidating the interaction between Aβ and membrane lipids could be critical in understanding potential pathways of Aβ toxicity, especially given the results of studies demonstrating that changes in membrane composition occur in AD [10], [11]. The two-dimensional liquid environments provided by lipid bilayers can profoundly alter protein structure and dynamics by both specific and nonspecific interactions. General physicochemical membrane properties, such as phase state, bilayer curvature, elasticity and modulus, surface charge, and degree of hydration can modulate these protein/lipid interactions; however, the exact chemical composition, i.e. extent of acyl chain unsaturation, conformation and dynamics of lipid headgroups and acyl chains, and protein–lipid selectivity arising from factors such as the hydrophobic matching at the protein–lipid interface of the membrane lipids also plays a role [12]. Importantly for misfolding diseases, these bilayer properties can not only modulate protein conformation, but also exert influence on oligomerization state, as numerous studies indicate substantial enhancement of protein and peptide aggregation in a membrane environment [13], [14], [15], [16], [17], [18], [19], [20]. Although lipid bilayers may act to induce aggregation and fibrillogenesis by providing environments that promote protein conformation and orientation conducive to assembly into protofibrillar and fibrillar structures [21], [22], [23], [24], cell membranes may be the direct target of amyloid forming peptides, resulting in cell death. This may be due to amyloid forming peptides inducing membrane permeabilization by altering bilayer structure [21], [24], [25], [26], [27] or by forming unregulated pore-like structures [28].

There are several point mutations associated with familial forms of AD. We were particularly interested in point mutations clustered around the central hydrophobic core of Aβ (E22G Arctic mutation, E22K Italian mutation, D23N Iowa mutation, and A21G Flemish mutation) (Fig. 1). With only the Flemish mutation being an exception, these missense mutations are not associated with increased Aβ secretion [29], [30], [31], [32], [33]. All of these Aβ variants, except the Flemish mutation, have a greater propensity to aggregate into protofibrils and/or fibrils than wild-type Aβ [31], [34], [35], [36], [37], and have been reported to be more toxic to neuroblastoma cells *in vitro* [38], [39], [40], [41], [42]. Interestingly, these mutations are located at the end of or directly adjacent to a sequence in Aβ (residues 16–21) that has been identified to have amyloidogenic properties [43]. Due to their clustering around positions 21–23, these mutations offer a unique opportunity to correlate toxicity to specific aggregate conformers, as point mutations alter the rates of Aβ aggregation, fibril formation, interaction with surfaces, and insertion into lipid bilayers by changing the hydrophobicity or charge following substitution. Here, we investigate how these point mutations alter the aggregation of Aβ in the presence of supported lipid bilayers.

## Results

### Free solution aggregation of Wild Type and mutant forms of Aβ results in similar fibril morphologies

**Figure 2:**
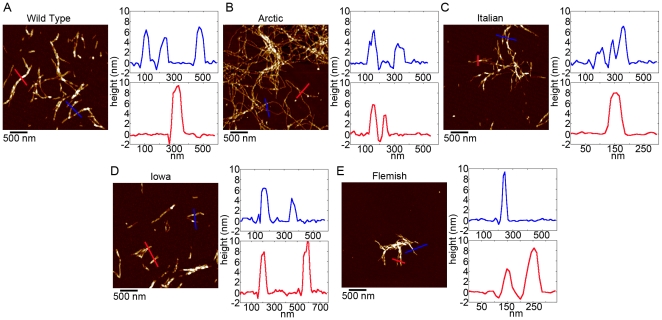
Wild Type and mutant Aβ fibril morphologies. A series of *ex situ* AFM images demonstrating the fibrillar morphologies associated with (A) Wild Type, (B) Arctic (C) Italian, (D) Iowa, and (E) Flemish Aβ aggregates. For all examples, color lines in the AFM image correspond to the profile of the same color presented to the right of each image.

To compare the resulting morphological features of fibrils formed by Wild Type and the mutant forms of Aβ under free solution conditions (without the presence of a lipid surface), 20 µM solutions of Wild Type, Arctic, Italian, Iowa, or Flemish Aβ (1–40) were incubated at 37^o^C and sampled at various times to check for the presence of fibrils. To control for the role of sample preparation and solution conditions, which have been shown to result in distinct polymorphic fibrillar structures of Wild Type Aβ [9], all samples were prepared via the same protocol [44]. While the presence of these specific mutations to alter the kinetics of Aβ has been demonstrated, we were interested if, once formed, there were distinct fibrillar morphologies associated with the different mutations. *Ex situ* AFM images and height profiles of fibrils formed by Wild Type, Arctic, Italian, Iowa, or Flemish Aβ (1–40) are shown in Figure 2. While the time required for fibril formation varied for Wild Type or mutant forms of Aβ, the final fibril structures formed under these conditions were indistinguishable based on gross morphological measurements. Fibrils formed from Wild Type or the mutant forms of Aβ (1–40) were 5–8 nm tall, had similar widths (within the error associated with the finite size and shape of the AFM tip), and often were twisted together when the fibril density was high.

### Wild Type Aβ (1–40) forms distinct aggregates on lipid membranes leading to membrane disruption

**Figure 3:**
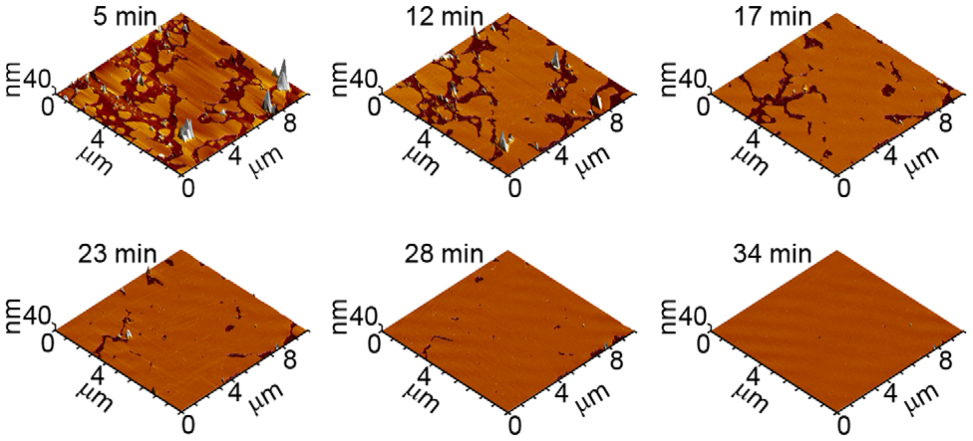
Formation of total brain lipid extract (TBLE) bilayer. A sequence of time-lapse *in situ* AFM images that demonstrate the formation of a supported TBLE bilayer on mica via vesicle fusion is shown. Initially, round bilayer patches appeared on mica as vesicles encountered the surface. With time, these patches gradually fused to form a defect-free bilayer patch, which provided an excellent model surface for studying the aggregation of Aβ and its mutant forms.

Supported bilayers on mica used in this study were produced through the fusion of total brain lipid extract (TBLE) vesicles (Fig. 3). The fusion of lipid vesicles to form supported planar bilayers is well-known [45]. It has also been documented that supported bilayers preserve many properties of free membranes such as lateral fluidity [46]. Furthermore, these TBLE bilayers provide an excellent model surface for studies aimed at elucidating the interaction of Aβ with lipids as they are comprised of a physiologically relevant ratio of membrane components, i.e. acidic and neutral phospholipids, gangliosides, cholesterol, sphingolipids, and isoprenoids. The formation of a supported bilayer is accelerated under the influence of the scanning AFM tip, resulting in the quick formation of defect-free model bilayers for use in these studies. Only defect-free TBLE bilayers, as determined by AFM images, were used to study Aβ/lipid interactions, and observations were limited to these verified defect-free regions. Once formed, the supported TBLE bilayers remained stable, i.e. there was no visible roughening or disruption, for 14–15 hours as assessed by AFM imaging.

**Figure 4:**
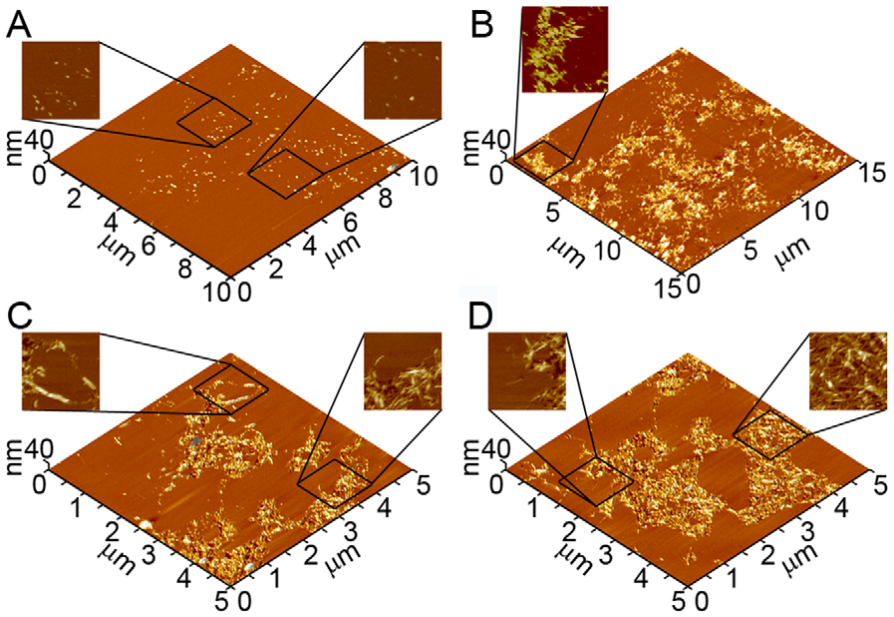
Representative *in situ* AFM images of Wild Type Aβ (1–40) aggregate formation on supported TBLE lipid bilayers. (A) 2–4 hours after injection of freshly prepared Wild Type Aβ (1–40), discrete oligomeric aggregates of Wild Type Aβ (1–40) appeared on the bilayer. Zoomed in examples of oligomers are shown. (B–D) After ∼9–12 hours, fibrils associated with vast regions of bilayer disruption were observed.

Experiments exposing defect-free TBLE bilayers to freshly prepared Wild Type Aβ (1–40) verified previously reported observations [16]. These experiments also served as controls for comparison with experiments conducted with mutant forms of Aβ (1–40). To control for any influence of the AFM tip on Aβ aggregation and Aβ-induced bilayer disruption, small areas of bilayer were intermittently scanned. Initially when TBLE bilayers were exposed to freshly prepared aliquots of a 20 µM solution of Wild Type Aβ (1–40) via injection into the fluid cell, there was little if any discernable deposition of protein onto the bilayer. With time (2–4 hours), discrete oligomeric aggregates of Wild Type Aβ (1–40) appeared on, or possibly in, the bilayer (Fig. 4A). These aggregates were stable and appeared to be relatively immobile, as they appeared unchanged in several successive AFM images taken over several hours. After ∼9–12 hours of exposure to Wild Type Aβ (1–40), the bilayer developed large regions of increased bilayer roughness, indicating disruption of the bilayer's structural integrity induced by the presence of Wild Type Aβ (1–40) (Fig. 4B–D). These areas of increased roughness were predominately associated with the presence of elongated, rigid fibrillar aggregates as evidence by their straight morphology. While the vast majority (∼95%) of observed fibrils was co-localized with distinctly disrupted bilayer surfaces, some fibrillar aggregates of Wild Type Aβ (1–40) were not associated with obvious adjacent areas of bilayer roughening.

### Point mutations in Aβ (1–40) alter the aggregate forms observed on lipid bilayers

**Figure 5:**
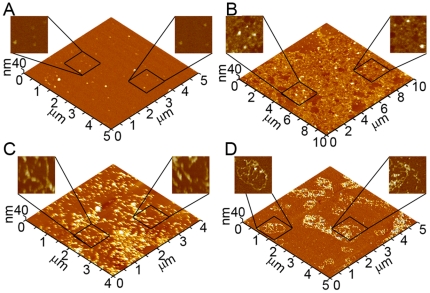
Representative *in situ* AFM images of Arctic Aβ (1–40) aggregate formation on supported TBLE lipid bilayers. (A) When TBLE bilayers were exposed to freshly prepared aliquots of 20 µM solution of Arctic Aβ (1–40), small oligomeric aggregates were observed within 1–2 hours. (B) After ∼6–8 hours, large regions of increased bilayer roughness were observed that appeared to be co-localized with discrete oligomeric aggregates. (C–D) Elongated fibrillar aggregates were also observed within 6–8 hours. (C) Many of these fibrils displayed straight morphologies. (D) These fibrillar aggregates often displayed enhanced curvature, forming large circular structures with many branching points.

When TBLE bilayers were exposed to a freshly prepared aliquots of 20 µM solution of Arctic Aβ (1–40), small oligomeric aggregates similar in morphology to those associated with Wild Type Aβ (1–40) (Fig. 5A) appeared on the lipid surface. These oligomers typically were observed within 1–2 hours after injection, exhibiting comparable stability and immobility to those formed from Wild Type. These oligomers also increased in number with time. Within ∼6–8 hours of exposure to Arctic Aβ, vast areas of increased bilayer roughness were observed; however, these areas were not associated with the co-localized rigid fibrillar aggregates as was the case with Wild Type (Fig. 5B). While these areas appeared to be populated by discrete oligomeric aggregates, there was a large number of morphologically indistinguishable oligomers that were not associated with disrupted bilayer structure. Elongated fibrillar aggregates were also observed within 6–8 hours of addition of Arctic Aβ (1–40) to the lipid bilayer. While many of these fibrils displayed straight morphologies reminiscent of those observed for Wild Type Aβ (1–40) (Fig. 5C), elongated aggregates displaying enhanced curvature were frequently observed for Arctic Aβ (1–40) (Fig. 5D). These highly curved extended aggregates often circled back upon themselves, forming large (100′s of nm wide) ring-like structures that were not associated with the formation of holes within the bilayer. These curved fibrillar structures also contained many branching points, which could also rejoin, resulting in the formation of small ring-like structures contained within the contour of the fibril itself.

**Figure 6:**
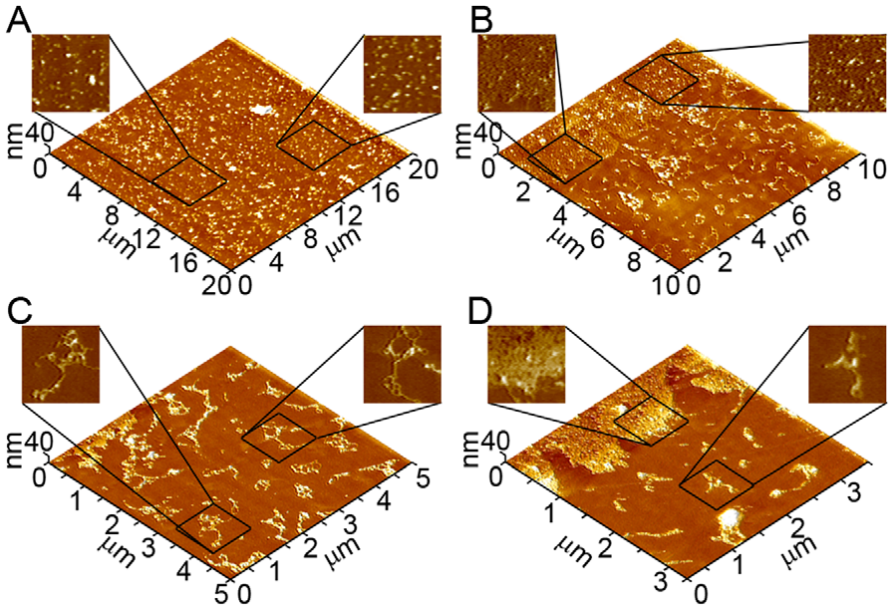
Representative *in situ* AFM images of Italian Aβ (1–40) aggregate formation on supported TBLE lipid bilayers. (A) Upon addition of a 20 µM solution of Italian Aβ (1–40) to the TBLE bilayer, oligomeric aggregates appeared within 2–4 hours. (B) After ∼10–12 hours of exposure to Italian Aβ (1–40), the bilayer developed large patches of increased bilayer roughness that often contained oligomeric aggregates. (B–D) Elongated fibrillar aggregates were observed within 8–10 hours of addition of Italian Aβ (1–40) to the lipid bilayer. These fibrillar aggregates displayed a variety of morphologies, predominantly displaying large curvature and branching.

We next investigated the interaction of Italian Aβ (1–40) with supported TBLE bilayers (Fig. 6). Upon exposure of a 20 µM solution of Italian Aβ (1–40) to the TBLE bilayer, oligomeric aggregates of similar morphology compared to those described for Wild Type and Arctic were observed on the bilayer surface within 2–4 hours (Fig. 6A). Again, these oligomers were stable and initially increased in number with time. After ∼10–12 hours of exposure to Italian Aβ (1–40), the bilayer developed large patches of increased bilayer roughness, corresponding to disruption of the bilayer's structural integrity (Fig. 6B). Similar to Arctic Aβ (1–40) as opposed to Wild Type, these areas of increased bilayer roughness were not clearly associated with rigid fibrils. However, elongated fibrillar aggregates were observed within 8–10 hours of addition of Italian Aβ (1–40) to the lipid bilayer (Fig. 6B–D). As was the case with Arctic Aβ, these fibrillar aggregates displayed a variety of morphologies. While several of these elongated aggregates of Italian Aβ (1–40) appeared rigid and lacking discernable curvature, the majority of fibrillar aggregates formed by Italian Aβ (1–40) had curled morphology reminiscent of the elongated aggregates observed for Arctic Aβ (1–40). These fibrils were often highly branched and contained a variety of large ring-like structures contained within their contour.

**Figure 7:**
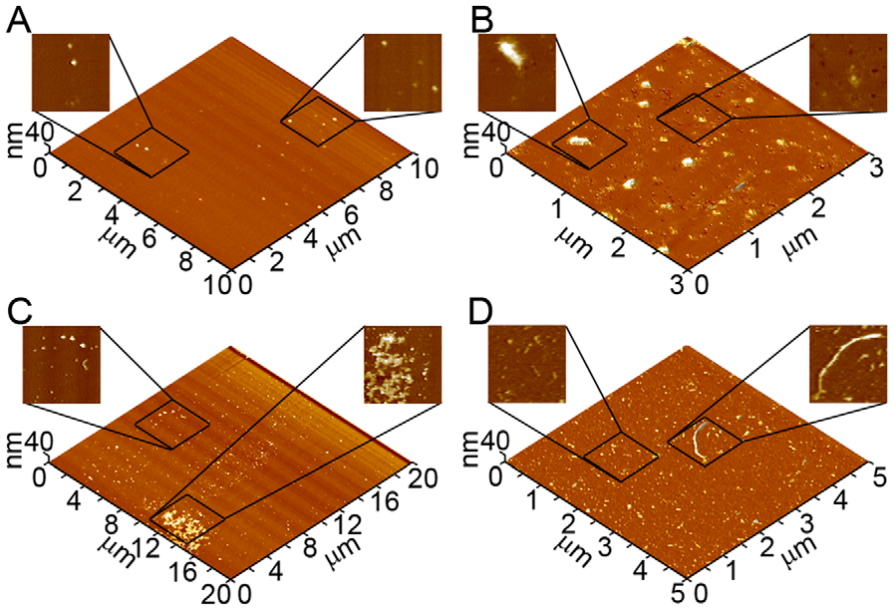
Representative *in situ* AFM images of Iowa Aβ (1–40) aggregate formation on supported TBLE lipid bilayers. (A) Upon exposure of a supported TBLE bilayer to a 20 µM solution of Iowa Aβ (1–40), a large number of discrete oligomeric aggregates appeared on the lipid surface within 2–3 hours. (B) Hole forming annular aggregates were observed after ∼6 hours. Larger amorphous aggregates formed from Iowa Aβ (1–40) were also present on the bilayer. (C) After ∼10–12 hours the bilayer developed small, discrete areas of disrupted lipid morphology. Many short putative fibrillar structures of Iowa Aβ (1–40) were also observed. (D) There were some larger fibrils formed on the bilayer that were morphologically very similar to the fibrils formed by Wild Type Aβ (1–40).

Upon exposure of a supported TBLE bilayer to a 20 µM solution of Iowa Aβ (1–40), a large number of discrete oligomeric aggregates appeared on the lipid surface within 2–3 hours (Fig. 7A). Like oligomers formed by Wild Type and other mutant Aβ (1–40) peptides, these oligomers were extremely stable and could be imaged for several hours. In some experiments with Iowa Aβ (1–40), hole forming annular aggregates were observed with a lip protruding ∼0.5 nm above the bilayer surface and an inner diameter of 32.7±4.5 nm (Fig. 7B). However, the inner diameters of these annular aggregates were much larger than would traditionally be considered a pore. While smaller, pore-like structures of Aβ have been reported in other studies [47], [48], these were the smallest annular structures we observed. After ∼10–12 hours of exposure to Iowa Aβ (1–40), the bilayer developed small, discrete areas of disrupted lipid morphology, i.e. enhanced roughness. These areas of enhanced roughness were much smaller than had previously been observed for Wild Type, Arctic, or Italian Aβ (1–40) (Fig. 7C). While these small areas of disrupted bilayer often had oligomeric structures contained within them, there was not a significantly larger number of oligomers associated with disruption areas compared to the number of oligomers on intact bilayer. Many short, putative fibrillar structures of Iowa Aβ (1–40) were observed (Fig. 7C). A few larger fibrils also appeared that were morphologically very similar to the fibrils formed by Wild Type Aβ (1–40) (Fig. 7D). These fibril structures did not appear to be associated with changes in the bilayer integrity. In spite of the appearance of fibrils and oligomers, abundant larger amorphous aggregates formed from Iowa Aβ (1–40) were also present on the bilayer (Fig. 7B). While these large amorphous aggregates extended higher above the bilayer surface in comparison with the previously described oligomers, they still appeared globular (round) in nature, making them distinct from fibrils. Despite this distinctly round morphology, these amorphous aggregates often had many smaller protofibril-like structures protruding from their periphery.

**Figure 8:**
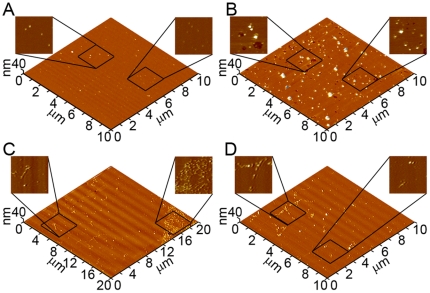
Representative *in situ* AFM images of Flemish Aβ (1–40) aggregate formation on supported TBLE lipid bilayers. (A) Within 3–5 hours after the injection of Flemish Aβ (1–40) into the fluid cell, oligomeric aggregates appeared on the bilayer surface. (B) Often small defects in the lipid bilayer that did not appear to be associated with aggregates would develop. There were also many large amorphous aggregates on the bilayer. (C) After ∼10–12 hours of exposure to Flemish Aβ (1–40), a few small patches of increased bilayer roughness associated with a high density of very short putative fibrillar aggregates developed. (D) Not all fibrils were associated with bilayer disruption.

When TBLE bilayers were exposed to freshly prepared aliquots of 20 µM solutions of Flemish Aβ (1–40) via injection into the fluid cell, oligomeric aggregates were not typically observed for 3–5 hours (Fig. 8A). Once oligomers appeared on the surface, they were stable for several hours. Often small bilayer defects developed (Fig. 8B), but these defects did not appear to have any Flemish Aβ (1–40) annular aggregates associated with their appearance like the annular hole forming aggregates observed for Iowa Aβ (1–40). After ∼10–12 hours of exposure to Flemish Aβ (1–40), supported bilayers developed a few small patches of increased bilayer roughness (Fig. 8C). These areas of increased roughness were predominately associated with a high density of very short putative fibrillar aggregates. However, not all fibrillar aggregates of Flemish Aβ (1–40) were associated with adjacent areas of bilayer disruption (Fig. 8D). The fibrils not associated with bilayer disruption tended to be isolated from other aggregate structures. In some experiments with Flemish Aβ (1–40), larger amorphous aggregates, similar to those formed by Iowa Aβ (1–40), were observed (Fig. 8B). Unlike those larger aggregates observed for Iowa Aβ (1–40), these amorphous aggregates did not appear to have smaller protofibril-like structures protruding from their periphery.

### Comparison of mutant Aβ aggregate size in the presence of lipid bilayers

**Figure 9:**
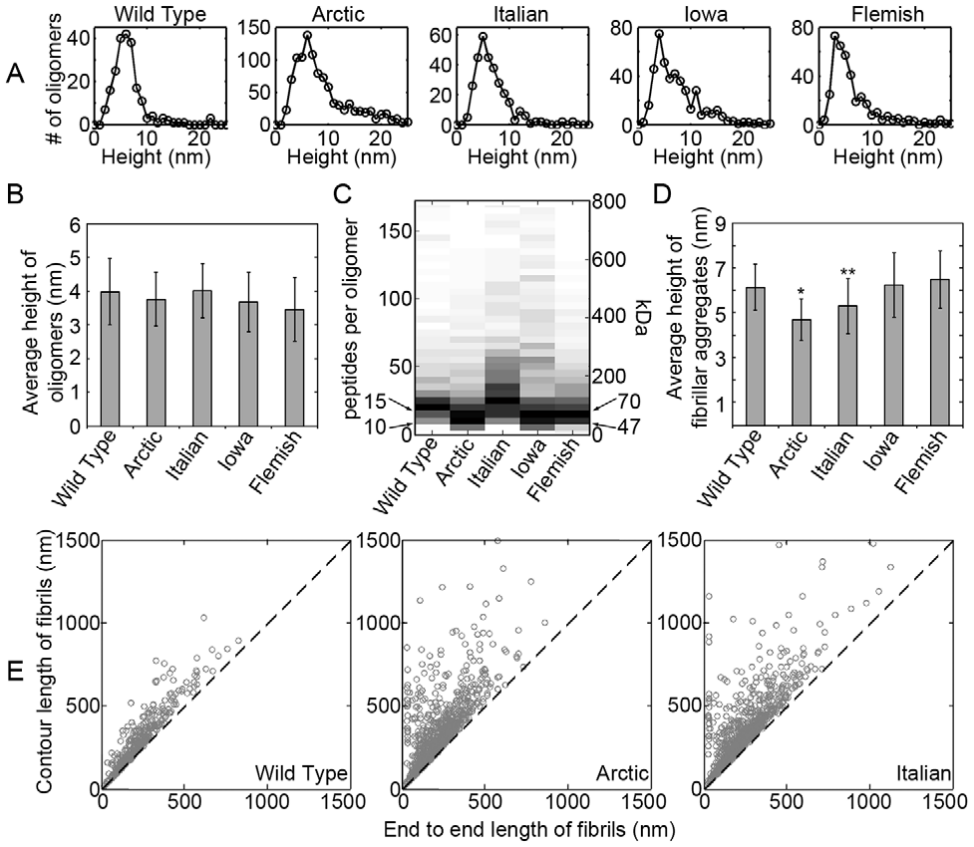
Quantification of morphological features of aggregates formed on TBLE bilayers by Wild Type or mutant forms of Aβ (1–40). (A) Histograms of height above the bilayer surface for oligomers formed by Wild Type, Arctic, Italian, Iowa, or Flemish Aβ (1–40) are shown. (B) When the average height above the bilayer surface of oligomers formed by Wild Type and the mutant forms were compared, oligomers were not significantly different as a function of mutation. (C) Based on corrected volume measurements and the molecular mass of Aβ (1–40), the numbers of peptides per oligomer and apparent mass of oligomers comprised of Wild Type, Arctic, Italian, Iowa, or Flemish Aβ (1–40) were calculated. The plots are color coded such that darker colors represent a greater abundance of oligomers composed of that number of molecules. Black arrows indicate where 10–15 peptides and 47–70 kDa oligomers would be observed. (D) The average height above the bilayer surface along the contour of elongated fibril aggregates comprised of Wild Type, Arctic, Italian, Iowa, or Flemish Aβ (1–40) are shown. Fibrils formed from Arctic (* indicates p<0.01) and Italian (** indicates p<0.05) Aβ (1–40) were significantly shorter compared to fibrils comprised of Wild Type Aβ (1–40). (E) Plots correlating the contour length to the end to end distance of fibrils formed from Wild Type, Arctic, or Italian Aβ (1–40) are shown. The dashed line represents the theoretical correlation for infinitely rigid rod-like structures.

We next compared measurable morphological features of oligomer and fibrillar aggregates of Wild Type and mutant forms of Aβ (1–40) formed in the presence of supported TBLE bilayers (Fig. 9). To obtain data sets of physical dimensions of only globular oligomers using our image analysis software, oligomers were defined as any feature protruding at least 2 nm above the bilayer surface with an aspect ratio (longest distance across to shortest distance across) less than 2.0, indicating a globular structure. Fibrils were defined as aggregates that protruded at least 3 nm above the bilayer surface with an aspect ratio greater than 2.5, indicating an elongated structure. These criteria were based on measurements of representative examples of each respective aggregate type. Measurements of oligomers and fibrils were compiled from multiple experiments and time points. The height (measured from the bilayer surface) distributions of globular oligomeric aggregates were not significantly different (based on a Spearman's rank correlation coefficient), when comparing Wild Type with each mutant form of Aβ (1–40), suggesting that oligomers formed from each mutation are structurally indistinguishable from those formed by Wild Type (Fig. 9A). The similarity between oligomer aggregates formed by Wild Type and the different mutant Aβ (1–40) peptides was further illustrated by comparison of the average height of oligomers formed by each type of Aβ (1–40) peptide (Fig. 9B). In an independent analysis, we used the corrected volume distributions of oligomers [49] to estimate the number of protein molecules per oligomer and the corresponding mass of oligomers formed by Wild Type, Arctic, Italian, Iowa, and Flemish Aβ (1–40). One caveat of this analysis is that it does not take into account any portion of the globular oligomer that may be inserted into the bilayer. Despite this limitation, the majority of oligomers formed by Wild Type and each mutant were in the range of 10–15 peptides per oligomer with a mass ranging from 47–70 kDa (Fig. 9C). These measured dimensions were within the range of a specific oligomeric structure (Aβ*56) that correlates with memory loss [50], and have been imaged by AFM [51]. Despite the majority of these oligomers being similar in size to Aβ*56, there was still a significant number of larger globular aggregates, particularly for the Italian and Iowa mutants, demonstrating that the population of oligomeric aggregates formed in the presence of lipid bilayers display considerable heterogeneity (Fig. 9C).

The average height above the surface along the contour of elongated, fibrillar aggregates was also determined (Fig. 9D). Unlike the average height of oligomers, when comparing the height of fibrils formed by mutant Aβ (1–40) peptides with those formed by Wild Type, fibrils comprised of Arctic (p<0.01) or Italian (p<0.05) Aβ (1–40) were significantly shorter based on a student's T-test (Fig. 9D). This suggests that fibrils formed by these two mutant forms of Aβ (1–40) are structurally different or are further inserted into the lipid bilayer when compared to those formed by Wild Type. Fibrils observed for Arctic and Italian Aβ (1–40) often appeared morphologically distinct to Wild Type fibrils as described above, which supports the notion that the fibrils are structurally unique. The primary morphological difference between fibrils formed by these different versions of Aβ (1–40) was an apparent increased curvature (or reduced persistence length) of the fibril structure. Therefore, we measured the contour and end to end distance of individual fibrils formed from Wild Type, Arctic, and Italian Aβ (1–40) on the TBLE bilayer. We then analyzed this data by constructing correlation plots of individual fibril contour length as a function of their end to end distance (Fig. 9E). For rigid structures with an infinitely large persistence length, the contour length would be equal to the end to end distance. For these infinitely rigid structures, the corresponding correlation plots would produce a line with a slope of 1, which is represented by the dashed line. As elongated fibril structures become less rigid (shorter persistence length), correlation between the contour length and end to end distance will deviate from this line. As the contour length increases, the spread of the correlation with end to end distance will increase with a decrease in persistence length. For the more rigid fibril structures observed for Wild Type Aβ (1–40) in the presence of lipid bilayers, the majority of data points for the correlation between contour length and end to end distance fell near the theoretical line for an infinitely rigid rod-like structure with very little spread (Fig. 9E). The correlation between contour length and end to end distance for fibrils comprised of Arctic Aβ (1–40) or Italian Aβ (1–40) formed in the presence of the bilayer deviated much more from the theoretical line with a large spread of data points with increased contour length. These features in the correlation plots indicate that fibrils formed from these two mutant Aβ (1–40) peptides were less rigid than their Wild Type counterparts, further supporting that these fibrils are structurally distinct. A feature of these plots is that for very short fibrils (contour length < persistence length) the data points fall very closely to the theoretical line of infinitely rigid rods. Due to this feature, analysis of fibrils formed from Iowa and Flemish Aβ (1–40) was inconclusive (data not shown) because the observed fibrils formed from these peptides tended to be very short (less than 200 nm in contour length).

### Comparison of induced lipid roughness by different point mutations of Aβ

**Figure 10:**
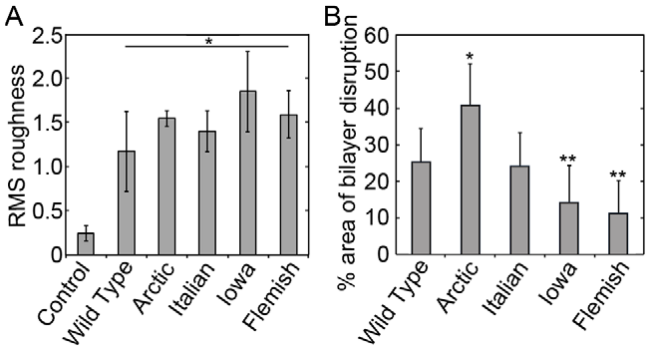
Quantification of bilayer roughening. (A) Quantitative assessment of bilayer disruption by analysis of root mean square (RMS) roughness of images taken before and after exposure to various Aβ (1–40) peptides is shown. Control corresponds to RMS roughness measurements taken on supported TBLE lipid bilayers that had not been exposed to any Aβ (1–40) peptides. Exposure to Wild Type, Arctic, Italian, Iowa, or Flemish Aβ (1–40) induced significant (* indicates p<0.01) roughening of the supported TBLE lipid bilayer. RMS roughness measurements of disrupted areas were restricted to areas that displayed enhanced roughness to prevent biased based on the extent of disruption. (B) The percent area of TBLE bilayers containing increased roughness induced by exposure to Wild Type, Arctic, Italian, Iowa, or Flemish Aβ (1–40) was measured from images obtained 10–12 hours after the initial injection of Aβ (1–40) peptides. Exposure to Arctic Aβ (1–40) resulted in a significantly larger area of the bilayer being disrupted in comparison to Wild Type (* indicates p<0.01). The extent of bilayer disruption was significantly reduced when bilayers were exposed to Iowa or Flemish Aβ (1–40) in comparison to Wild Type (** indicates p<0.05).

We next used image processing software to analyze the extent of bilayer disruption. A freshly formed bilayer had a root mean square (RMS) surface roughness of 0.24±0.09 nm measured over a total of 148 µm^2^ (Fig. 10A). To prevent error associated with the size of the regions of disrupted bilayer structure, RMS roughness measurements of areas that had been destabilized by Aβ (1–40) and mutant Aβ (1–40) peptides were restricted only to portions of the images containing disrupted regions, excluding regions of the bilayer not altered by the presence of Aβ (1–40) from the analysis. RMS roughness measurements are also highly dependent on the coverage of Aβ (1–40) aggregates present on the surface; therefore, aggregate structures were filtered out of the analysis. All RMS measurements were taken from images representing at least 10–12 hours of bilayer exposure to Wild Type or mutant forms of Aβ (1–40). After 10 hours of exposure to Wild Type Aβ (1–40), disrupted portions of the bilayer surface displayed significantly (p>0.01) enhanced RMS surface roughness increasing to 1.18±0.46 nm measured over a total of 56.7 µm^2^ (Fig. 10A). Likewise, significant (p>0.01) bilayer roughening occurred after 10 hours of exposure to all mutant Aβ (1–40) peptides (1.55±0.19 nm measured over 1,579 µm^2^, 1.41±0.24 nm measured over 896 µm^2^, 1.86±0.46 nm measured over 117 µm^2^, 1.60±0.27 nm measured over 381 µm^2^ for Arctic, Italian, Iowa, and Flemish respectively). Despite different aggregate types (or lack thereof) being associated with regions of bilayer disruption for Wild Type or mutant forms of Aβ (1–40), there was no significant differences in the measured RMS roughness of these regions when comparing each mutant peptide to Wild Type.

Although the resulting surface roughness associated with bilayer disruption by Wild Type or mutant forms of Aβ (1–40) was similar in magnitude after 10 hours of exposure, the fraction of the bilayer surface area exhibiting some amount of increased roughness varied (Fig. 10B) (which is one reason why the RMS roughness measurements reported above were taken over different sized areas). For Wild Type Aβ (1–40), 25.4±9.0% of the bilayer surface area exhibited increased surface roughness. The Arctic mutation significantly (p<0.01) increased the percent area of bilayer disruption (41.0±10.9%) after 8 hours of co-incubation. Despite resulting in morphologically distinct fibrillar aggregates, the Italian mutation did not result in a significantly different percent of bilayer surface area with increased roughness (24.3±9.4%) in comparison to Wild Type. Both the Iowa (14.32±10.0%) and Flemish (11.2±9.4%) mutations resulted in a significantly (p<0.01) smaller percentage of the bilayer being disrupted in comparison to Wild Type.

## Discussion

In this study we compared the interaction of Wild Type, Arctic, Italian, Iowa, and Flemish Aβ (1–40) peptides with supported TBLE lipid bilayers with a focus on aggregate morphology and bilayer disruption. Point mutations have been shown to promote protein aggregation by destabilizing the native, globular state of a protein [52]. While it has already been demonstrated that these specific point mutations alter the rate of Aβ aggregation [29], [30], [31], [32], [33], [51], [53], [54], we demonstrate here that these mutations result in a heterogeneous mixture of aggregate species and polymorphic fibrillar structures, especially in the presence of lipid bilayers. Furthermore, the ability of Aβ to disrupt the structural integrity of bilayers is notably modulated by these mutations.

Results presented here highlight the potential role electrostatic and hydrophobic properties of Aβ play in its ability to bind, insert, and potentially disrupt lipid membranes. The Arctic mutation replaces a polar, negatively charged glutamic acid with a nonpolar, neutral glycine. The Arctic mutation also represents a change in the hydropathy index [55] from −3.5 to −0.4, increasing the hydrophobic nature of the peptide. We speculate that the resultant increased hydrophobic nature increases the insertion of Arctic Aβ (1–40) once bound to the bilayer surface leading to enhanced bilayer disruption. The observed enhanced bilayer disruption at micromolar concentrations is consistent with recent coarse-grained molecular dynamic simulations of fibril-forming amphipathic peptides in the presence of lipid vesicles suggesting that the ongoing process of aggregation leads to the development of defects in the bilayer surface, underlying the ability of mutations that enhance fibrillogenesis to appear more toxic [18]. The Italian mutation replaces a polar, negatively charged glutamic acid residue with a polar, positively charged lysine, with a corresponding small decrease in hydrophobic character (hydropathy index changes from −3.5 to −3.9). Despite the Italian mutation being associated with an increased rate of aggregation [41], the extent of bilayer disruption did not significantly change in comparison with Wild Type Aβ (1–40). However, fibril morphologies observed for the Italian mutation in the presence of the lipid bilayer (as will be discussed in more detail later) were morphologically distinct compared to Wild Type fibrils. The Iowa mutation replaces a polar, negatively charged aspartic acid with a polar, neutral asparagine; however, there is no change in the hydrophobic character of the peptide based on the hydropathy index. As the Iowa mutation does not alter the hydrophobic character of Aβ (1–40), the reduced ability of the Iowa mutation to disrupt supported lipid bilayers appears to be related to eliminating a negative charge from the peptide. The Arctic mutation also resulted in the elimination of a negative charge from Aβ (1–40); however as previously stated, the Arctic mutation resulted in increased bilayer disruption due to its increased hydrophobic character. The Flemish mutation replaces a nonpolar, neutral alanine residue with a nonpolar, neutral glycine, leading to a large decrease in hydrophobic character (a change of 1.8 to −0.4 in the hydropathy index). As there is no net change in peptide charge, the decreased extent of aggregation and bilayer disruption observed for the Flemish mutation is most likely a result of its increased hydrophilic nature. Interestingly, it has often been observed that exogenously added Aβ will selectively bind a subset of hippocampal neurons and neuroblastoma cells in culture [56], [57]. These selective interactions could potentially be regulated by not only the composition of the cellular membrane but also by the electrostatic and hydrophobic properties of Aβ, which is supported by the altered aggregation patterns observed for different mutant peptides.

The central peptide region, where the mutations studied here are located, has been implicated as being important in Aβ aggregation and fibril structure. EPR studies of Aβ fibril structure using site-directed spin-labeling demonstrated that this central region is sandwiched between two more highly ordered regions in the fibril structure [58]. Later studies using systematic proline replacement in Aβ (1–40) demonstrated that residues 22 and 23 probably occupy turn positions within the fibril structure [59]. Interestingly, these mutations are located at the end of or directly adjacent to a sequence in Aβ (residues 16–21) that has been identified to be highly amyloidogenic [43], indicating that this region may be a viable target for preventing Aβ aggregation for therapeutic purposes. In this regard, several antibodies and single chain variable fragments specific for this region of Aβ appear to prevent aggregation and in some cases reduce toxicity [60], [61], [62]. As each mutation alters aggregation kinetics as well as AD phenotype, each may have different propensities to form various abnormal structures like spherical oligomers, and this may be heavily influenced by the chemical environment or liquid/solid interfaces. Observations reported here support the notion that this region plays a role in lipid/Aβ interactions that can influence aggregate morphology and the ability of Aβ to disrupt bilayer integrity. In particular, the propensity to form polymorphic fibrillar structures on lipid bilayers appears to be heavily influenced by point mutations in this region.

A striking feature of many amyloid forming peptides is their ability to form more than one stable fibril structure [4]. The fibrillar aggregates formed by the Arctic and Italian Aβ (1–40) peptides in the presence of the supported lipid bilayers appeared to be distinct polymorphs compared to those formed by the Wild Type peptide. This observation suggests that electrostatic and hydrophobic interactions with membrane surface can induce polymorphic aggregate structures of Aβ. In the case of the Arctic mutation, it has been reported that aggregates (oligomer, protofibrils, and fibril) formed by this mutation are indistinguishable to those formed by Wild Type under free solution conditions [51]. However, the aggregation process and resulting aggregate morphology of Arctic differed dramatically in comparison to Wild Type in the presence of the anionic surface of mica, pointing to the ability of surfaces to influence aggregate structure [51]. This ability of surfaces to alter aggregate morphology of amyloidogenic peptides has been well documented for a variety of systems [63], [64]. It appears that the chemical environments provided by lipid membranes have the ability to structurally influence the aggregation of Aβ into elongated structures. The two mutations that led to shorter, less rigid fibril structures both occur at the 22^nd^ residue of Aβ. In both cases, a negative charge is removed, indicating that this residue may experience specific electrostatic interactions with lipids that influence its ability to form distinct fibrillar polymorphs. However, it appears that the exact composition of the bilayer and the peptide preparation protocol could also play a role in this interaction, as the more highly curved fibril structures have been observed for Wild Type Aβ (1–40) on TBLE bilayers by other investigators [15]. The Iowa and Flemish mutations did not appear to result in different morphologies of fibrils compared to Wild Type; however, the extent of fibril formation appeared to be reduced in the presence of the bilayer by both of these mutations. As it has been proposed that different polymorphs may be associated with variations in AD phenotype [9], it is tempting to speculate that local chemical environments of lipid membranes may influence the aggregate state of Aβ. Furthermore, the propensity of mutant Aβ to form different polymorphic aggregates may underlie the phenotypic variations associated with these mutations.

## Materials and Methods

### Sample preparation

Synthetic Wild Type, Arctic, Italian, Iowa, and Flemish Aβ (1–40) peptides were obtained from AnaSpec Inc. (San Jose, CA). All peptides were prepared in the same manner [44]. In short, peptides were treated with Hexafluoroisopropanol (HFIP) to dissolve pre-existing aggregates. The HFIP was evaporated off in a Vacufuge concentrator (Eppendorf), resulting in a small peptide film. These peptide films were dissolved in dimethyl sulfoxide (DMSO) to make a 2000 µM stock solution. These stock solutions were then dissolved directly into 37°C PBS buffer (pH 7.3) to a final concentration of 20 µM (final DMSO concentration of 1%).

Total brain lipid extract (porcine) was purchased from Avanti Polar Lipids (Alabaster, AL) in a lypholized state and was resuspended in PBS (pH 7.3) at a concentration of 1 mg/ml. Using an acetone/dry ice bath, bilayers and multilayer lipid sheets were formed by five cycles of freeze-thaw treatment [15], [16]. The lipid suspensions were then sonicated for 15 minutes to promote vesicle formation. All experiments were performed with the same lot of lipids.

### AFM imaging conditions

In situ AFM experiments were performed with a Nanoscope V MultiMode scanning probe microscope (Veeco, Santa Barbara, CA) equipped with a sealable fluid cell and a closed-loop vertical engage J-scanner. Images were taken with V-shaped oxide-sharpened silicon nitride cantilever with a nominal spring constant of 0.5 N/m. Scan rates were set at 1 to 2 Hz with cantilever drive frequencies ranging from ∼8–10 kHz. Concentrated TBLE vesicle solution was added to the cell in 40 µL aliquots by the hanging drop method and allowed to fuse *in situ*. Once a 40×40 µm patch of defect-free bilayer was formed, the cell was flushed to remove vesicles remaining in solution. Only defect-free bilayers that were 40×40 µm were used for studies with Aβ. Next, 40 µL of 20 µM of freshly prepared Aβ (1–40) solution in PBS was added via the channels in the fluid cell.

### Quantitative analysis of AFM images

Image analysis of all AFM images was performed using Matlab equipped with the image processing toolbox (Mathworks, Natick, MA). Physical dimensions of most aggregates were measured automatically in this way: 1) Images were imported into Matlab. 2) Images were flattened to correct for curvature due to the imaging process. 3) Flattened images were converted into binary maps of aggregate locations by using a height threshold (set at ∼1 nm). This was accomplished by assigning values of 1 to any pixel of the image that represented a height above the threshold and assigning a value of 0 to any pixel corresponding to a height below the threshold. 4) The binary map was used to locate aggregates within the original AFM image using pattern recognition algorithms. 5) Once a discreet aggregate was located, dimensions (including height, volume, average diameter, width, length, aspect ratio, position within the image, etc.) were automatically measured. Each aggregate was also assigned an individual number so that aggregates chosen based on specific measured properties could be located, allowing us to verify that chosen dimensions corresponded to specific aggregate types. In this way large data sets were automatically constructed that could be used to keep track of thousands of individual aggregates and sorted based on specific dimensional characteristics. Based on inspection of individual representative aggregate types and verified using our tracking system, we assigned specific dimensional characteristics to specific aggregate types. Oligomers were defined as 2–10 nm in height with an aspect ratio (longest distance across to shortest distance across) less than 2, indicating a globular structure. Fibrils were defined as aggregates greater than 3 nm in height that had an aspect ratio greater than 2.5. For determination of fibril contour length and end to end distance, individual objects in an AFM image were identified by height thresholding and filtering based on aspect ratio. The software then incorporated a fast parallel thinning algorithm [65] to create a pixel skeleton for each object present in the AFM image. Once the fibril skeleton was obtained, the endpoints of each skeleton were determined and used to calculate end to end distance. The pixel skeleton was used to determine contour length and average height along contour of the fibril.

### Determining the number of peptides per oligomer from AFM images

Volume measurements were partially corrected for error associated with the finite size of the AFM probe based on geometric models [49]. The volume of an individual Aβ peptide was estimated based on its molecular weight and the average density of proteins [66], [67]. By dividing the observed corrected volume of each individual aggregate by the estimated volume of a single monomer, the number of molecules per each oligomer was calculated. This calculation assumes perfect packing of individual monomers within the oligomer and that the density of the proteins is the same in aggregated and unaggregated forms.
